# Versatile vector tools for efficient protein screening across multiple expression systems

**DOI:** 10.1002/2211-5463.70262

**Published:** 2026-06-10

**Authors:** Zhimin Zhu, Yaqing Liu, Lei Qin, Feng Yu, Qisheng Wang, Yahui Liu

**Affiliations:** ^1^ Shanghai Institute of Applied Physics Chinese Academy of Sciences Shanghai China; ^2^ College of Light Industry and Food Engineering Guangxi University Nanning China; ^3^ Department of Radiology, Tongji Hospital, Tongji Medical College Huazhong University of Science and Technology Wuhan China; ^4^ Shanghai Synchrotron Radiation Facility, Shanghai Advanced Research Institute Chinese Academy of Sciences Shanghai China; ^5^ National Key Laboratory of Non‐food Biomass Energy Technology Institute of Biology, Guangxi Academy of Sciences Nanning China

**Keywords:** eukaryotic expression systems, high‐throughput screening, multi‐host protein expression, vector toolkit

## Abstract

Heterologous protein expression is a cornerstone of biological research, but screening across different expression systems is often labor‐intensive. We developed versatile vector tools for E. coli, insect, and mammalian cells, offering His, MBP, and GST tags. These tools feature a standardized interface that enables rapid vector switching via homologous recombination, requiring only a single pair of primers for PCR amplification. This significantly accelerates gene construction, thereby greatly increasing the efficiency of parallel protein screening. We demonstrate successful protein expression and purification in various systems using these tools, showcasing their ability to streamline protein screening and improve efficiency. Our results highlight the versatility and effectiveness of these vector tools in facilitating rapid protein screening across multiple expression systems.

## Graphical abstract

A unified vector toolkit enables rapid protein expression screening across E. coli, insect, and mammalian cells. A single primer pair amplifies the target gene, which is inserted into any vector via a standardized interface. This streamlined workflow eliminates repeated cloning steps, accelerating the identification of optimal expression conditions for challenging recombinant proteins.

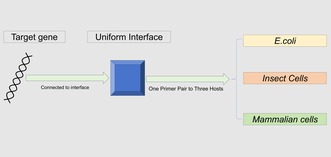

**Abbreviations**
3C: Human Rhinovirus 3C Protease cleavage site
E1: Ubiquitin‐activating enzyme
E2: Ubiquitin‐conjugating enzyme
GST: Glutathione S‐Transferase
HRV 3C: Human Rhinovirus 3C Protease cleavage site
Huwe1: HECT, UBA, and WWE domain‐containing protein 1
IMAC: Immobilized Metal Affinity Chromatography
MBP: Maltose‐Binding Protein
PEI: Polyethylenimine
SDS/PAGE: Sodium Dodecyl Sulfate/Polyacrylamide Gel Electrophoresis

The heterologous expression of proteins in recombinant host systems is a fundamental methodology for biomedical research and biotechnology, enabling the study of protein function and the production of therapeutics and reagents [1, 2]. The primary goal was to obtain sufficient yields of soluble, correctly folded protein, a process that remains largely empirical and often requires testing across multiple expression platforms [3, 4, 5, 6, 7, 8].

Escherichia coli is a workhorse for protein production due to its simplicity, speed, and low cost [9, 10, 11, 12]. However, its prokaryotic biology often fails to support the proper folding and essential post‐translational modifications of complex eukaryotic proteins [13, 14]. Eukaryotic systems, such as baculovirus‐infected insect cells and mammalian cells, address these limitations by providing more sophisticated folding environments and modification machinery, but at the expense of increased cost, time, and technical complexity [15, 16]. Consequently, identifying the optimal expression system for a given protein is a critical, yet often protracted, screening process.

This screening bottleneck is exacerbated by the lack of standardized molecular tools. Conventional workflows require the re‐cloning of a target gene into separate, host‐specific vectors for each trial—a process involving unique primer sets, multiple PCRs, and distinct assembly strategies. This serial approach consumes significant time and resources, hindering rapid iteration and optimization.

To overcome this methodological constraint, we developed a unified vector toolkit for parallel protein screening across E. coli, insect, and mammalian expression systems. The core innovation is a standardized “uniform interface,” a common DNA sequence (GTTCTGTTCCAGGGGCCCGGATCCTAAAAGCTTGTCGAGAAGTACTAGAGGA) engineered into all vectors. This interface allows a single PCR‐amplified gene fragment, generated with one universal primer pair, to be seamlessly inserted via homologous recombination into any vector within the kit. The toolkit incorporates multiple affinity tags (8 × His, MBP, GST) to facilitate purification and enhance solubility. Here, we describe the design, validation, and application of these versatile vectors, demonstrating their utility in accelerating the expression and purification of proteins across diverse hosts, thereby streamlining a foundational workflow in molecular biology (Fig. 1).

**Fig. 1:**
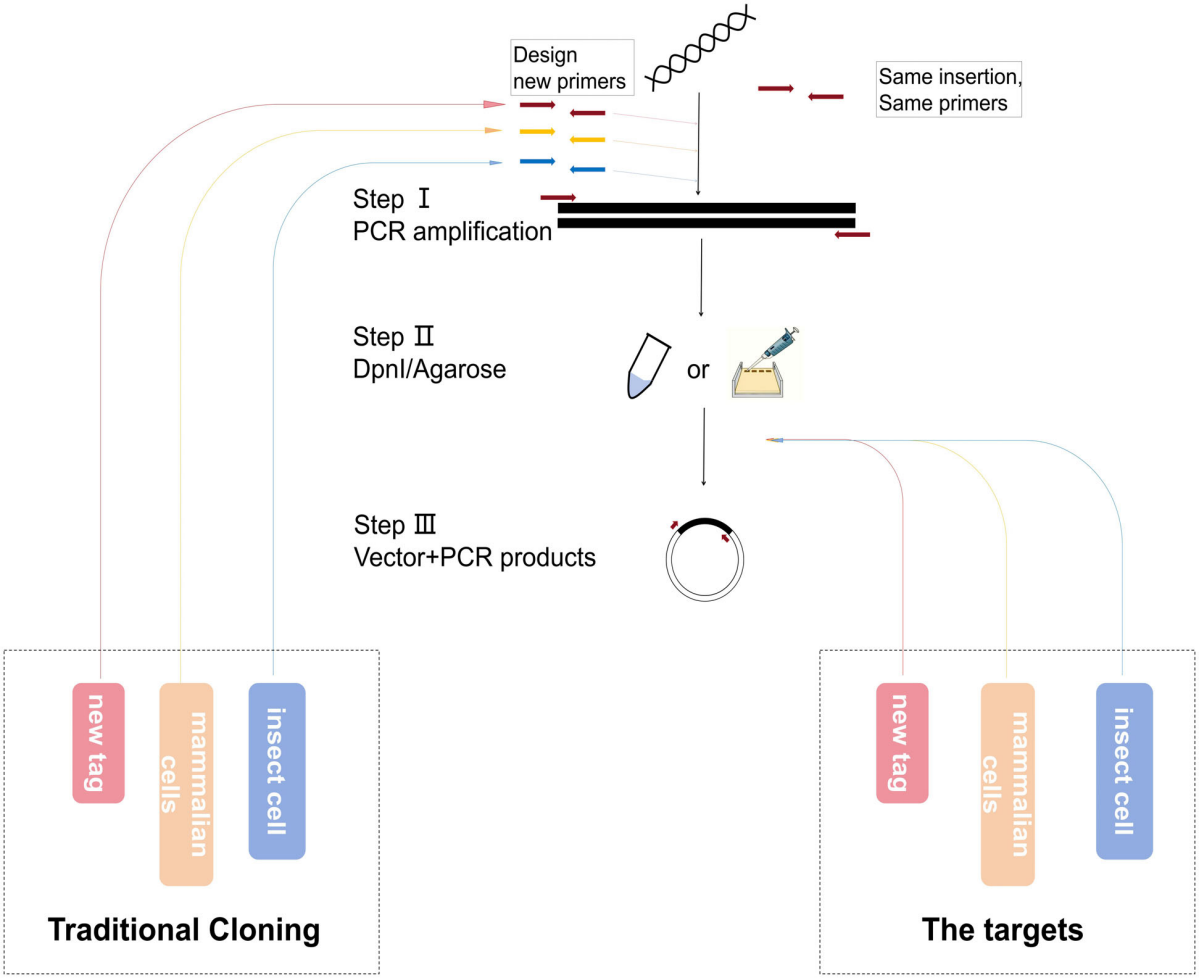
Comparison of traditional expression plasmid screening methods with our protein rapid screening vector tools. Traditional screening methods require multiple PCRs for different vectors. For the fragment preparation (step II) and the Gibson Assembly of vector and insert step (III), they are identical in both traditional and unified interface workflows.

## Materials and methods

### Vector design

**Fig. 2:**
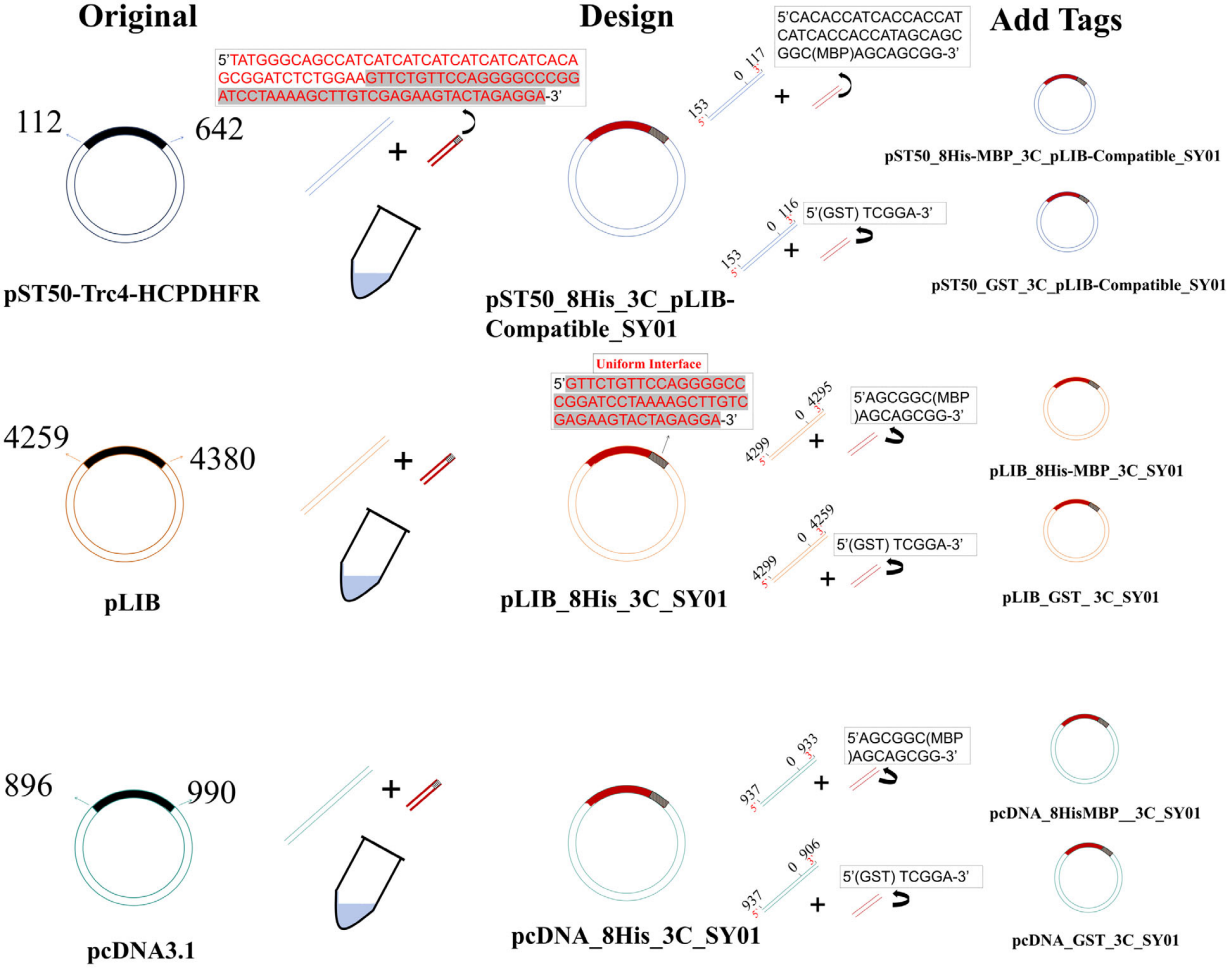
Design of the vector kit. Vectors were constructed using the method of homologous recombination.

The following expression vectors were selected for modification: *E. coli* expression vector pST50Trc4‐HCPDHFR, insect cell expression vector pLIB, and mammalian cell expression vector pcDNA3.1 (Invitrogen) (Fig. 2). For pST50‐Trc4‐HCPDHFR, the genomic region from nucleotides 112 to 642 was replaced by TATGGGCAGCCATCATCATCATCATCATCATCACAGCGGATCTCTGGAAGTTCTGTTCCAGGGGCCCGGATCCTAAAAGCTTGTCGAGAAGTACTAGAGGA through homologous recombination. This resulted in the creation of the pST50_8 × His_3C_pLIB‐Compatible_SY01 vector. Similarly, in the other two vectors, the region 4259‐ATCCCG…TAGAGGA‐4380 of pLIB and the region 896‐CTAGCG…CTCGAG‐990 of pcDNA3.1 were replaced by the same insert sequence mentioned above. This resulted in the pLIB_8 × His_3C_SY01 vector and pcDNA_8 × His_3C_SY01 vector, respectively. Furthermore, the insert sequence includes a ‘uniform interface’, (GTTCTGTTCCAGGGGCCCGGATCCTAAAAGCTTGTCGAGAAGTACTAGAGGA), which is central to the rapid screening workflow. The MBP and GST tags are incorporated into three newly designed vectors, respectively. The MBP tag was inserted downstream of 8 × His tag, through a (SSG) linker with the HRV 3C site, which offers numerous advantages, including low‐temperature activity, high specificity, and proven utility. In contrast, the GST tag replaced the 8 × His tag and was linked to the HRV 3C site by an SD linker. All new vectors were constructed using homologous recombination, and successful construction was confirmed by Sanger sequencing. The Gibson cloning procedure was performed using the ClonExpress II one‐step cloning kit (Vazyme, China) according to manufacturer's instructions. The full‐length sequences of all the vectors are presented in Appendix S1.

#### Vector construction via homologous recombination

The core vectors of this toolkit (pST50_8 × His_3C_pLIB‐Compatible_SY01, pLIB_8 × His_3C_SY01, and pcDNA_8 × His_3C_SY01) were constructed using a PCR‐based homologous recombination strategy, as illustrated in Fig. 2. The following generic protocol was applied to each backbone vector (pST50Trc4‐HCPDHFR, pLIB, and pcDNA3.1):

1. Primer Design: Two long primers (Forward Insertion Primer and Reverse Insertion Primer, approximately 60–80 nucleotides (nt)) were designed for each vector. Each primer consisted of:
  - A 5′ terminus with 20–25 nt of homology to the target insertion site in the linearized vector backbone.
  - A central region encoding the desired insert sequence (the ‘uniform interface’, an 8 × His tag, an HRV 3C protease site, and relevant linkers).
  - A 3′ terminus with 20–25 nt of homology to the other end of the vector backbone breakpoint, enabling circularization.
2. Backbone Linearization: The parental vector was linearized by inverse PCR using primers that annealed back‐to‐back, amplifying the entire plasmid except for the region to be replaced.
3. Insert Generation: The complete DNA cassette containing the new genetic elements (e.g., tag, protease site, and interface) was generated as a single, double‐stranded DNA fragment by PCR using the long homologous recombination primers described in Step 1. This PCR product was purified.
4. Homologous Recombination Assembly: The purified linearized vector backbone (50–100 ng) and the insert fragment (with a molar ratio of insert:vector of approximately 2:1) were mixed. The assembly was performed using the ClonExpress II one‐step cloning kit (Vazyme, China) according to the manufacturer's instructions. The reaction mixture was incubated at 37 °C for 30 min.
5. Transformation and Screening: The assembled product was transformed into competent *E. coli* DH5α cells. Positive clones were selected on antibiotic‐containing plates and screened by colony PCR using primers flanking the insertion site. All final constructs were verified by Sanger sequencing across the entire modified region.

### Cloning of target genes into expression vectors

The gene sequences encoding the E1 ubiquitin‐activating enzyme‐related protein (GenBank: XM_003875809.1), E2 ubiquitin binding enzyme‐related proteins (GenBank: XM_003877863.1), and huwe1(GenBank:XP_005262022.1)were searched on GenBank. The sequences were synthesized by Sangon Biotech (Shanghai, China) and used as templates for PCR amplification. The forward and reverse primers for the E1 putative ubiquitin‐activating enzyme, E2 ubiquitin‐conjugating enzyme, and Huwe1^3568−4374^ are listed in Table 1. The PCR products were purified by gel extraction. The E1 ubiquitin‐activating enzyme and E2 ubiquitin‐conjugating enzyme related protein genes were ligated into the pST50_8 × His‐MBP_3C_pLIB‐Compatible_SY01 vector. Similarly, Huwe1^3568−4374^ was ligated into pST50_8 × His‐MBP_3C_pLIB‐Compatible_SY01, pLIB_8 × His_MBP_3C_SY01, and pcDNA_8His_3C_SY01 vectors. Once the recombinant plasmids were constructed, they were transformed into *E. coli* DH5α cells. Antibiotic‐free liquid LB culture medium was then added to the transformed cells, which were subsequently incubated for 1 h in a 37 °C shaker. Thereafter, the cells were evenly spread on LB agarose plates containing 0.1 mg/mL ampicillin antibiotic and grown overnight in a 37 °C incubator. The following day, single clones were selected for continued culture expansion, and the plasmids were extracted using a DNA extraction kit after the OD_600_ of the bacterial solution reached approximately 0.8, thereby obtaining a substantial number of recombinant plasmids.

**Table 1:** Primers of recombined gene PCR.

E9AWN6	Forward primer	GTTCTGTTCCAGGGGCCCGGATCCATGCTCAACGCGGCCCT
	Reverse primer	ATCCTCTAGTACTTCTCGACAAGCTTTTATTAGAGACCATGCCCGCACAG
E9B2J3	Forward primer	GTTCTGTTCCAGGGGCCCGGGATCCATGAGCGGCGCAGGCAAC
	Reverse primer	ATCCTCTAGTACTTCTCGACAAGCTTTTATCAAAGATCAATCTCCTC
Huwe1^3568−4374^	Forward primer	GTTCTGTTCCAGGGGCCCGGGATCCAAGATGGGTGTCCTCTGGC
	Reverse primer	ATCCTCTAGTACTTCTCGACAAGCTTTTAGGCCAGCCCAAAGCCTTC

### Gene expression

The recombinant plasmids of E1 putative ubiquitin‐activating enzyme, E2 ubiquitin‐conjugating enzyme, and Huwe1^3568−4374^ were transformed into *E. coli* BL21(DE3) competent cells and plated on LB agar plates containing 0.1 mg/mL ampicillin. The clones were selected and cultured in LB liquid medium containing 0.1 mg/mL ampicillin for expansion in a shaker at 37 °C. Once the OD600 of the bacterial solution reached 0.8, IPTG was added to a final concentration of 0.1 mm and genes expression was induced overnight at 18 °C.

The pLIB‐Huwe1^3568−4374^ recombinant plasmid was constructed and subsequently transformed into *E. coli* DH10Bac receptor cells. This allowed Huwe1^3568−4374^ to be transposed into the bacmid within DH10Bac competent cells. Successfully integrated clones were identified using blue‐white colony screening, and the recombinant bacmid DNA was purified using a plasmid recovery kit.

The recovered Bacmid was transfected into Sf9 cells to generate baculovirus encoding the Huwe1^3568−4374^ recombinant protein. P1 viral stock was collected, and 500 mL (1.8 × 10^6cells/ml) of Sf9 cells were transfected with 2.5 mL of viral stock, and cultured for 72 h in a shaker at 27 °C at 130 rpm to enable recombinant protein expression.

To express pcDNA_8 × His_3C_SY01‐Huwe1^3568−4374^ using the pcDNA3.1 vector in a transient system with PEI and 293F cells, a PEI stock solution was first prepared by dissolving PEI in a buffer, sterile filtering, and storing at −20 °C. 293F cells were maintained in SMM 293‐TII Expression Medium (SinoBiological, China) at 37 °C with shaking. On the day of transfection, DNA‐PEI complexes were prepared by mixing diluted pcDNA_8 × His_3C_SY01‐Huwe1^3568−4374^ plasmid DNA with diluted PEI solution and incubate for 30 min. The DNA‐PEI complexes were then added to the cells at a density of 3 × 10^6^ cells/mL, and the cells were incubated at 37 °C with shaking. After 36 h, the culture was diluted with fresh medium and incubated for an additional 48 h. The cells were then harvested by centrifugation for subsequent protein purification and analysis.

### Protein purification

Bacterial and insect cell cultures were centrifuged at 2000 rpm for 10 min, and the supernatant was poured off. The pellet was resuspended in buffer A (30 mm Tris–HCl 8.0, 300 mm NaCl, 5% glycerol) using a homogenizer. The cells were lysed using a high‐pressure cell disruptor at 700 bar for *E. coli* and 900 bar for both insect cells and 293F cells, with a breakage time for 10 min. The lysate was centrifuged at 15000 rpm for 50 min, and the supernatant was collected. The supernatant was purified using Ni‐NTA gravity column, and the protein was eluted with buffer A containing 15 mm, 30 mm and 300 mm imidazole, respectively. The eluate from 300 mm imidazole fraction was subsequently subjected to further purification by size‐exclusion chromatography, using buffer B (30 mm Tris–HCl 8.0, 100 mm NaCl, 5% glycerol, 2 mm DTT) for elution. The eluted protein samples were analyzed by SDS/PAGE.

## Results

### Introduction to protein rapid screening vector tools

**Fig. 3:**
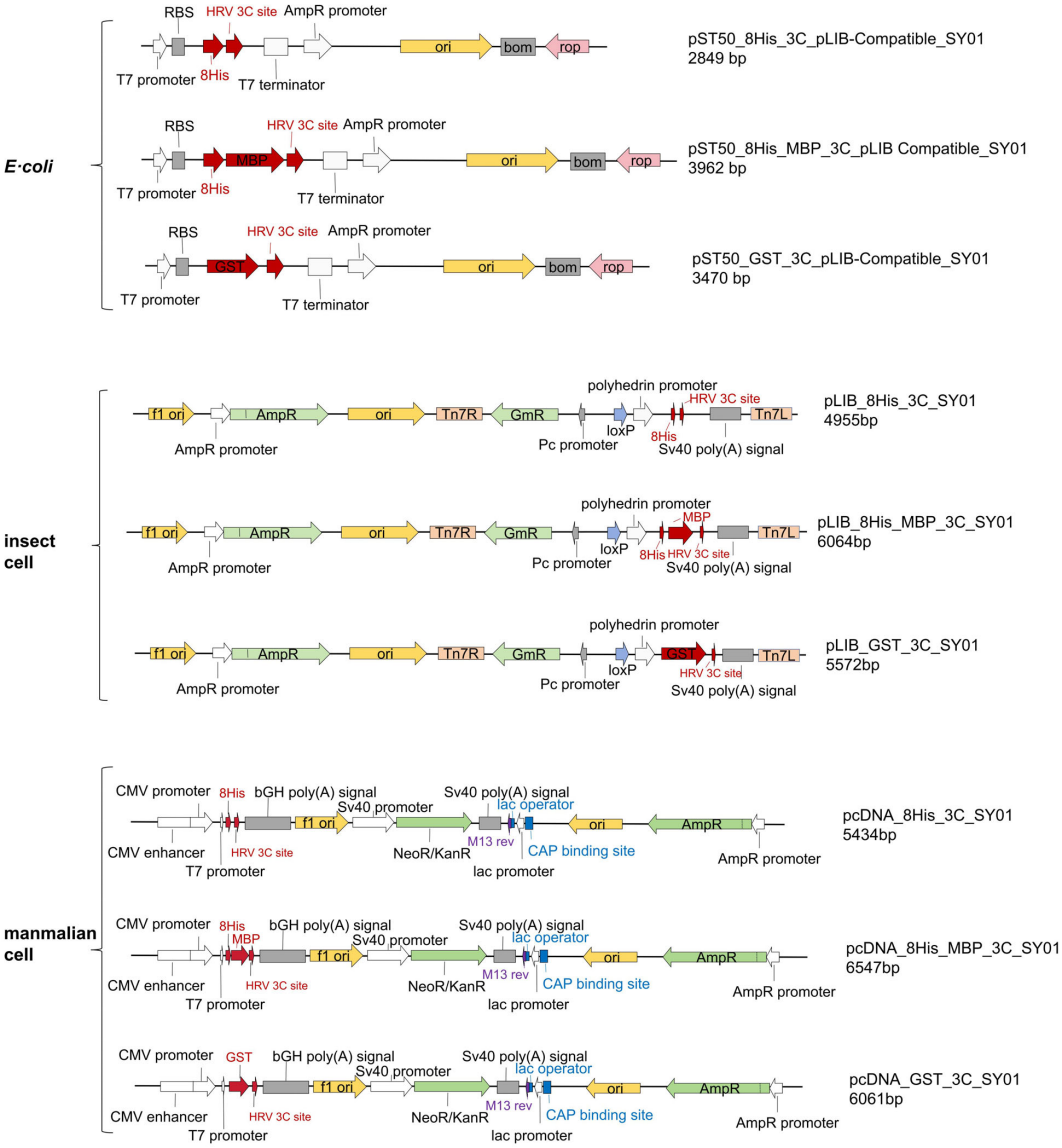
This vector kit supports three expression systems (*E. coli* expression system, insect cell expression system and mammalian cell expression vector). In each expression system, three purification tag options—8 × His, 8 × His‐MBP, and GST—are available for fusion to the target protein. Therefore, this vector kit comprises nine expression vectors in total.

The expression vector pST50Trc4‐HCPDHFR [17], pLIB [18], and pcDNA3.1 were selected as the backbone templates of bacterial expression system, insect cell expression system and mammalian cell expression system, respectively. These three vectors are commonly used in their respective expression systems and meet the requirements for most protein expression studies. Purification tags, such as the MBP‐tag and GST‐tag, can also enhance solubility and facilitate purification. This vector kit supports three expression systems: *E. coli* expression system, insect cell expression system, and the mammalian cell expression vector. In each expression system, the target protein can be tagged with one of three purification tags: 8 × His tag, 8 × His‐MBP tag, or GST tag. Therefore, this vector kit includes nine expression vectors. Finally, we designed the following vectors: pST50_8 × His_3C_pLIB‐Compatible_SY01 (2849 bp), pST50_GST_3C_pLIB‐ Compatible_SY01 (3470 bp), pST50_8 × His‐MBP_3C_pLIB‐Compatible_SY01 (3959 bp), pLIB_8 ×His_3C_SY01 (4955 bp), pLIB_GST_3C_SY01 (5572 bp), pLIB_8 × His‐MBP_3C_SY01 (6064 bp), pcDNA_8 × His_3C_SY01 (5434 bp), pcDNA_8 × His_MBP_3C_SY01 (6547 bp), and pcDNA_8 × His_GST_3C_SY01 (6061 bp) (Fig. 3).

In addition to the features listed above, the key feature of this Protein Rapid Screening Vector Tool is its ‘uniform interface’ (GTTCTGTTCCAGGGGCCCGGATCCTAAAAGCTTGTCGAGAAGTACTAGAGGA). Because of this “uniform interface”, it is only necessary to use the same pair of primers. F: GTTCTGTTCCAGGGGCCCGGATCC_(followed by the start codon and gene‐specific sequences), R: TCCTCTAGTACTTCTCGAC AAGCTT (GGATCC optional) TTA (followed by the reverse complement of stop codon and gene‐specific sequences) for one PCR amplification of the target gene. The amplification product obtained with any vector from our set of vector tools has overlapping ends. Gibson assembly can be used to quickly assemble the target gene with the required vector. Gibson cloning procedure was performed using the ClonExpress II one‐step cloning kit (Vazyme, China) according to the manufacture's instructions. For this Protein Rapid Screening Vector Tools, if the expression system or purification tag needs to be changed, the same PCR products can be assembled with the new vector, without re‐designing primers and PCR amplification.

### Recombinant expression and purification of ubiquitin‐conjugating enzyme in *E. coli* cells

**Fig. 4:**
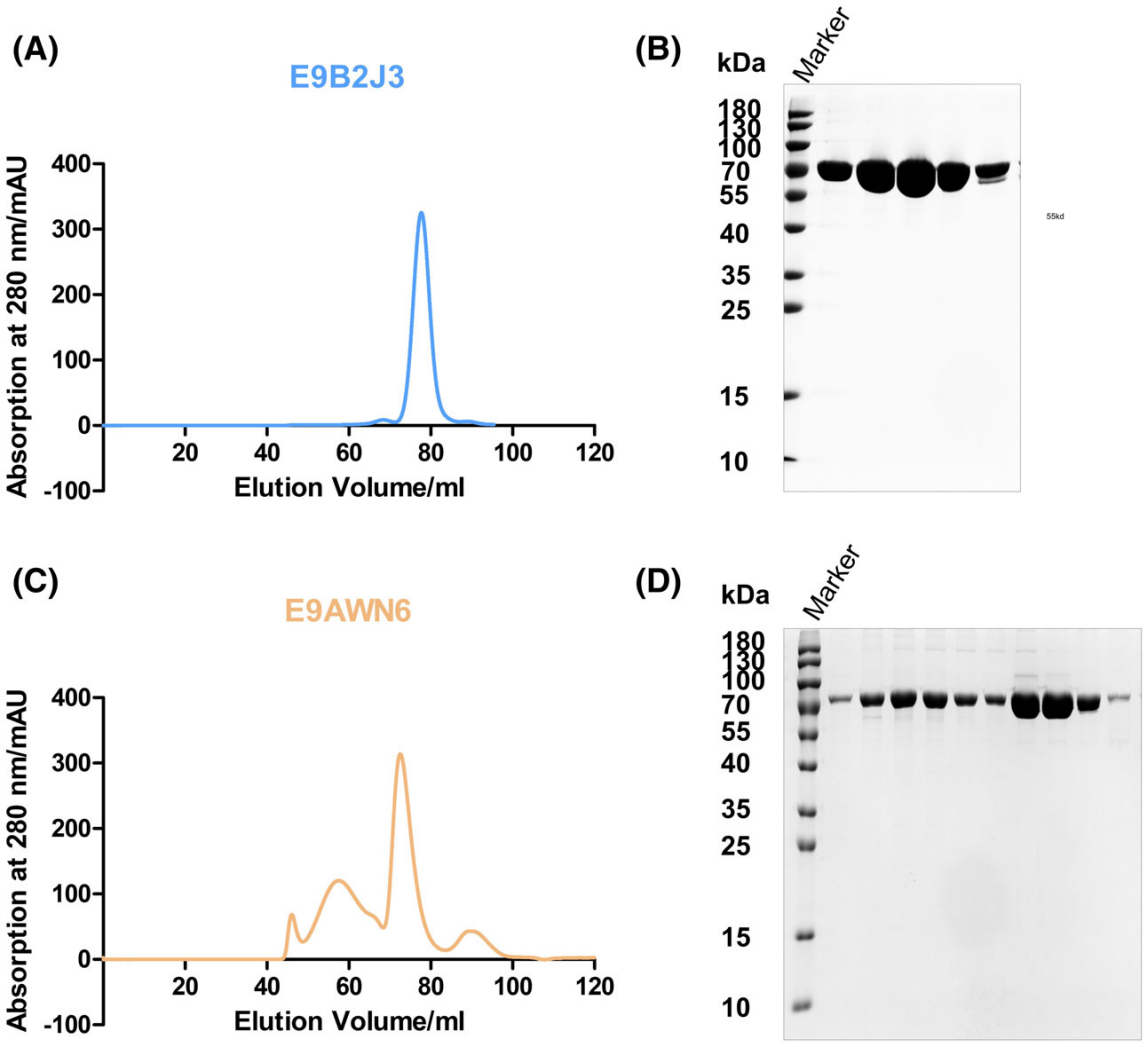
Purification results of E2 ubiquitin‐conjugating enzyme (E9B2J3), E1 putative ubiquitin‐activating enzyme (E9AWN6) genes in *E. coli*. (A) Size‐exclusion chromatography result for E9B2J3, (B) SDS/PAGE result for E9B2J, (C) size‐exclusion chromatography result for E9AWN6, (D) SDS/PAGE result for E9AWN6.

To confirm the utility of the newly constructed vectors, we inserted the genes for E1 putative ubiquitin‐activating enzyme and E2 ubiquitin‐conjugating enzyme of Leishmania mexicana into the pST50_8 × His‐MBP_3C_pLIB‐Compatible_ SY01 vector. The pST50_8 × His‐MBP_3C_pLIB‐Compatible_SY01‐Putative ubiquitin‐activating enzyme (E9AWN6) and the pST50_8 × His‐MBP_3C_pLIB‐Compatible_SY01‐E2 recombinant plasmids of ubiquitin‐conjugating enzyme (E9B2J3) were transferred into *E·coli* BL21 (DE3) cells. After the firstround purification, the respective recombinant proteins with MBP tags were obtained and further polished by size exclusion chromatography. The eluted protein samples were prepared and separated by 10% (w/v) polyacrylamide gels. The results are presented in Fig. 4. E9B2J3 eluted as a single peak on a Superdex 200 column (Fig. 4A). The highest peak for E9AWN6 eluted at approximately 78 mL in the size‐exclusion chromatography (Fig. 3A), with an estimated molecular weight of approximately 70 kDa (Fig. 4B). The highest peak of the E9AWN6 was observed at approximately 73 mL in the size‐exclusion chromatography (Fig. 4C), with the protein size on the gel estimated to be approximately 75 kDa (Fig. 4D). This finding is consistent with the expected size. Taken together, these findings demonstrate that the recombinant plasmid encoding the ubiquitin‐conjugating enzyme‐constructed using our newly developed vector—was successfully replicated, and the target gene it carries was efficiently expressed, yielding the encoded recombinant protein after purification. Our newly constructed multifunctional cloning vector is a viable option.

### Purification of HUWE1^3568^ ^−4374^ recombinant protein

**Fig. 5:**
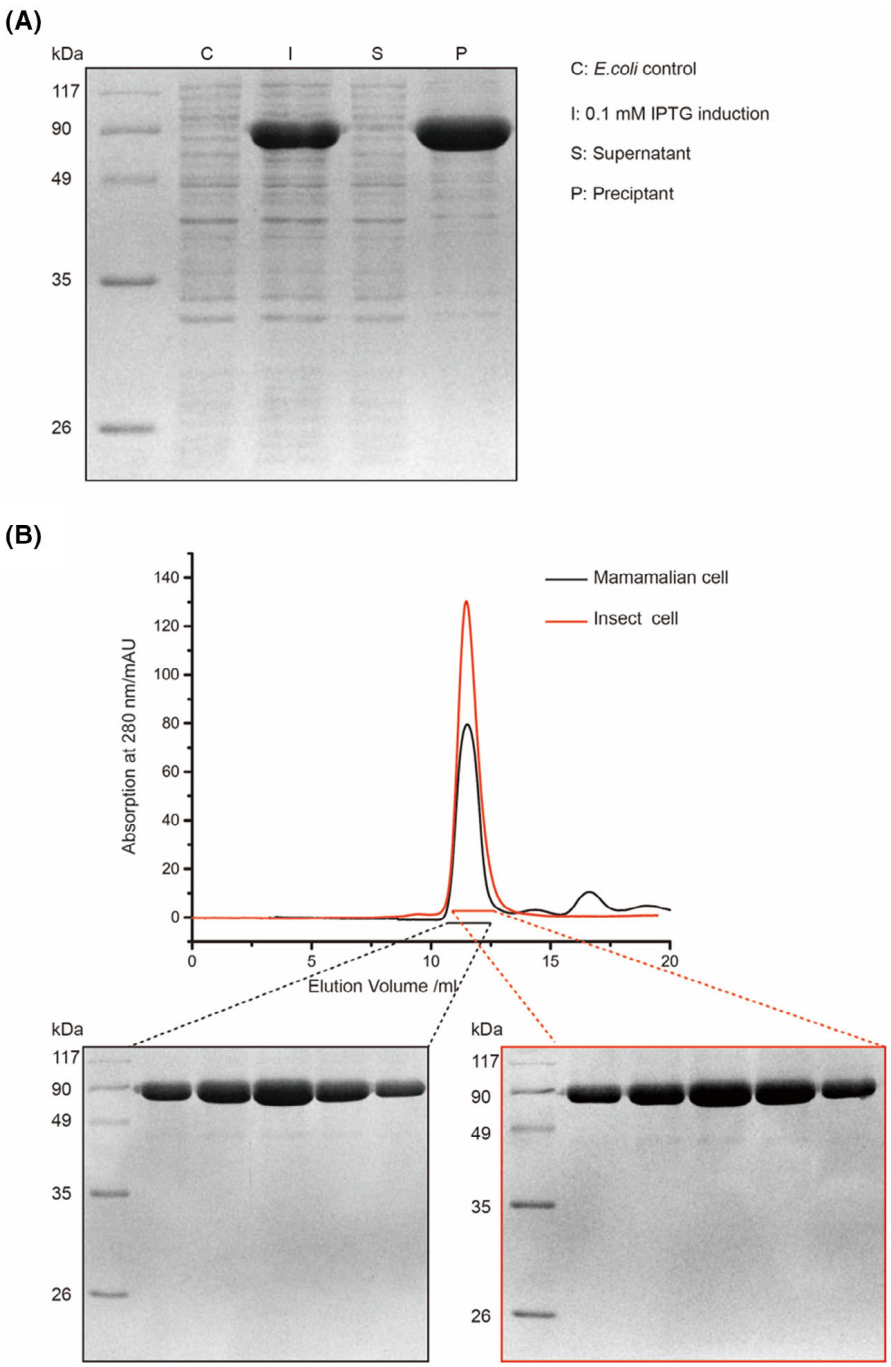
Expression and Purification of HUWE1^3568−4374^ gene in *E. coli*, insect cells and mammalian cells. (A) Expression of 8 × His‐Huwe1^3568−4374^ in *E. coli* resulted in inclusion body formation. (B) Size‐exclusion chromatography and SDS/PAGE analysis of 8 × His‐HUWE1^3568−4374^ expressed in both insect cells and mammalian cells, demonstrating successful purification. The data demonstrate successful cross‐host expression testing using our streamlined cloning method.

Initially, an *E. coli* expression system was employed to attempt to express Huwe1^3568−4374^. The pST50_8 × His _3C_pLIB‐Compatible_SY01‐Huwe1^3568−4374^ expression plasmid was introduced into *E. coli* BL21(DE3) cells. The bacteria were cultured in LB medium, and induced overnight with 0.1 mm IPTG at low temperature. Following SDS/PAGE analysis, the protein was expressed exclusively as inclusion body (Fig. 5A). Therefore, the pLIB_8 × His_3C_SY01‐Huwe1^3568−4374^ and pcDNA_8 × His_3C_SY01‐Huwe1^3568−4374^ plasmids were generated and expressed in Sf9 cells and 293F cells. After amplification and collection of the cell pellet, the cells were subjected to a series of purification steps. The target protein with a His‐tag was purified using affinity chromatography and size‐exclusion chromatography (Fig. 5B), and the purity was confirmed by SDS/PAGE, and the protein migrated at the expected molecular weight (Fig. 5B). It was demonstrated that the target gene encoding Huwe1^3568−4374^ was successfully expressed in both insect cells and mammalian cells.

## Discussion

The design strategy involved first selecting a suitable vector for each expression system, and then adding a ‘uniform interface’ and purification tags to facilitate expression purification. The expression vectors pST50Trc4‐HCPDHFR [17], pLIB [18], and pcDNA3.1 were selected as the backbone templates for the bacterial expression system, insect cell expression system, and mammalian cell expression system, respectively. A Gibson assembly ‘uniform interface’ sequence and a purification tag (8 × His/GST/MBP) were inserted into these backbone templates. To obtain the target protein without the purification tag, an HRV 3C proteinase site was also inserted between the purification tag and the target protein.

The development of the Protein Rapid Screening Vector Tool provides a useful addition to the existing molecular biology and protein engineering toolkit. This tool addresses the common challenges associated with heterologous protein expression, such as the need for the labor‐intensive process of vector replacement for multiple expression systems. By providing a ‘uniform interface’ and a variety of purification tags, our vector kit simplifies and accelerates the process of protein screening.

The successful expression and purification of target proteins in *E. coli*, insect cells, and mammalian cells using our vector tools demonstrate the versatility and efficiency of this system. The ability to use the same pair of primers for PCR amplification across different vectors significantly reduces the time and effort required for gene construction. This innovation is particularly beneficial for researchers who need to screen multiple proteins across various expression systems, as it streamlines the workflow and reduces costs.

Moreover, the inclusion of different purification tags (8 × His, MBP, and GST) allows for flexibility in protein purification and enhances the solubility and yield of the expressed proteins. This feature is crucial for obtaining high‐quality proteins for structural and functional studies, as well as for industrial applications. While the current toolkit utilizes a His‐tag for unified purification, the modular design readily accommodates the future incorporation of alternative affinity tags, such as the Twin‐Strep tag, to suit specific experimental needs for mild elution or enhanced purity.

While the toolkit offers significant flexibility, it is important to acknowledge a practical limitation of the unified interface approach: the performance of affinity tags is not universally equivalent across different host systems. As comprehensively reviewed elsewhere [19, 20], tag choice often requires optimization depending on both the target protein and the expression host. For instance, polyhistidine‐based IMAC purification can copurify endogenous metal‐binding proteins, a challenge that may be more pronounced in complex eukaryotic lysates. Therefore, while our vector set streamlines initial screening, optimal tag selection and purification conditions may still require host‐dependent refinement.

Beyond single‐gene expression, the modular architecture of the uniform interface system provides a straightforward path for adapting these vectors for co‐expression applications. A key strategy to improve the solubility and yield of challenging proteins is to co‐express them with binding partners or molecular chaperones. To enable this, the vector backbone can be engineered to include additional expression cassettes, each flanked by a compatible interface sequence. For example, a second uniform interface could be introduced upstream or downstream of the primary cassette, allowing for the sequential or simultaneous assembly of multiple genes into a single vector, creating a polycistronic operon analogous to systems such as pST44 in *E. coli* [17]. This modification would allow researchers to rapidly test the effect of various binding partners across different host systems using the same streamlined cloning principle demonstrated here for single genes.

The vector toolkit is designed for cost‐effectiveness and simplicity, utilizing a ‘uniform interface’ that allows the use of the same primers for PCR amplification across different vectors, significantly reducing time and costs associated with reordering primers and performing additional PCRs. This system is particularly advantageous for researchers working with multiple genes and expression systems. In contrast, Gateway cloning, a recombination‐based method, offers high throughput and versatility, allowing the simultaneous insertion of multiple DNA fragments into a single vector. However, it requires proprietary enzyme mixes (BP Clonase and LR Clonase), which can be expensive. Golden Gate cloning, which uses type IIS restriction enzymes, enables the assembly of multiple DNA fragments in a single reaction with high efficiency and precision, making it ideal for constructing complex genetic circuits. However, it requires careful design of overhangs and may not be as straightforward as the our vector toolkit system for routine cloning tasks.

The ability to rapidly screen and optimize protein expression in different systems can accelerate the pace of research in various fields, including drug discovery, biotechnology, and synthetic biology. For instance, the efficient production of recombinant proteins can facilitate the development of new therapeutics and vaccines, as well as the study of complex biological processes.

## Conclusion

This study presents the development of a comprehensive set of vector tools designed to streamline the process of rapid protein screening across multiple expression systems, including *E. coli*, insect cells, and mammalian cells. The vector kit, which features a “uniform interface” and a variety of purification tags (8 × His, MBP, and GST), enables efficient and cost‐effective gene construction and protein expression. The results obtained demonstrate the successful expression and purification of target proteins in different systems, thus emphasizing the utility and flexibility of these vector tools.

The innovative design of using the same pair of primers for PCR amplification across different vectors has been shown to significantly reduce the time and labor involved in protein screening. This approach is particularly advantageous for researchers working with multiple genes and expression systems, as it simplifies the process of vector replacement and protein expression optimization.

In conclusion, the vector tools developed in this study offer a practical contribution to the field of protein expression. These tools offer a practical solution to the challenges associated with heterologous protein production, providing a versatile and efficient method for rapid protein screening. Future research will focus on further validating these tools with a broader range of proteins and exploring additional applications in both basic research and industrial biotechnology.

## Conflict of interest

The authors declare no conflicts of interest.

## Author contributions

YL and ZZ conceived and designed the project. YL and LQ performed the experiments and acquired the data. ZZ and YL analyzed and interpreted the data. QW provided essential resources and materials. ZZ wrote the original draft of the manuscript. FY reviewed and edited the manuscript. YL supervised the project. All authors have read and approved the final version of the manuscript.

## Supporting information


**Appendix S1.** Full length of all vectors.

## Data Availability

The datasets used and/or analyzed during the current study available from the corresponding author upon reasonable request.
